# Supplementary material for: Classification of Skin Cancer Using Novel Hyperspectral Imaging Engineering via YOLOv5
Source: J Clin Med. 2023 Feb 1;12(3):1134. doi: 10.3390/jcm12031134 (PMC9918106; doi:10.3390/jcm12031134)
Supplement: Supplementary file 1 [file jcm-12-01134-s001.zip › jcm-2190947-supplementary.pdf]

Article

Classification of Skin Cancer Using Novel Hyperspectral Imaging Engineering via YOLOv5: Supplementary Material

Hung-Yi Huang [1], Yu-Ping Hsiao [2,3], Arvind Mukundan [4], Yu-Ming Tsao [4], Wen-Yen Chang [5,*] and Hsiang-Chen Wang [4,6,7,*]

1. Department of Dermatology, Ditmanson Medical Foundation Chiayi Christian Hospital, Chiayi 60002, Taiwan
2. Department of Dermatology, Chung Shan Medical University Hospital, No. 110, Sec. 1, Jianguo N. Rd., South District, Taichung City 40201, Taiwan
3. Institute of Medicine, School of Medicine, Chung Shan Medical University, No. 110, Sec. 1, Jianguo N. Rd., South District, Taichung City 40201, Taiwan
4. Department of Mechanical Engineering, Advanced Institute of Manufacturing with High Tech Innovations (AIM-HI) and Center for Innovative Research on Aging Society (CIRAS), National Chung Cheng University, No. 168, University Rd., Min Hsiung, Chiayi 62102, Taiwan
5. Department of General Surgery, Kaohsiung Armed Forces General Hospital, No. 2, Zhongzheng 1st Rd., Lingya District, Kaohsiung 80284, Taiwan
6. Director of Technology Development, Hitspectra Intelligent Technology Co., Ltd., 4F, No. 2, Fuxing 4th Rd., Qianzhen District, Kaohsiung 80661, Taiwan
7. Department of Medical Research, Dalin Tzu Chi General Hospital, No. 2, Min-Sheng Rd., Dalin Town, Chiayi 62247, Taiwan

* Correspondence: wenyen85@gmail.com (W.-Y.C.); hcwang@ccu.edu.tw (H.-C.W.)

Abstract: This document provides the supplementary material for the article “Identification and Classification of Skin Cancer using Novel Hyperspectral Imaging Engineering”. The first section explains about the YOLO Architecture and the second section describes the definition of the classification indicators used in this study.

Keywords: skin cancer; hyperspectral imaging; convolutional neural network; YOLO

S1. YOLO Architecture and Loss Functions

The network is mainly composed of backbone, neck, and head. Backbone is a convolutional neural network layer, neck is a feature extraction layer, and Head uses the GIOU loss function.

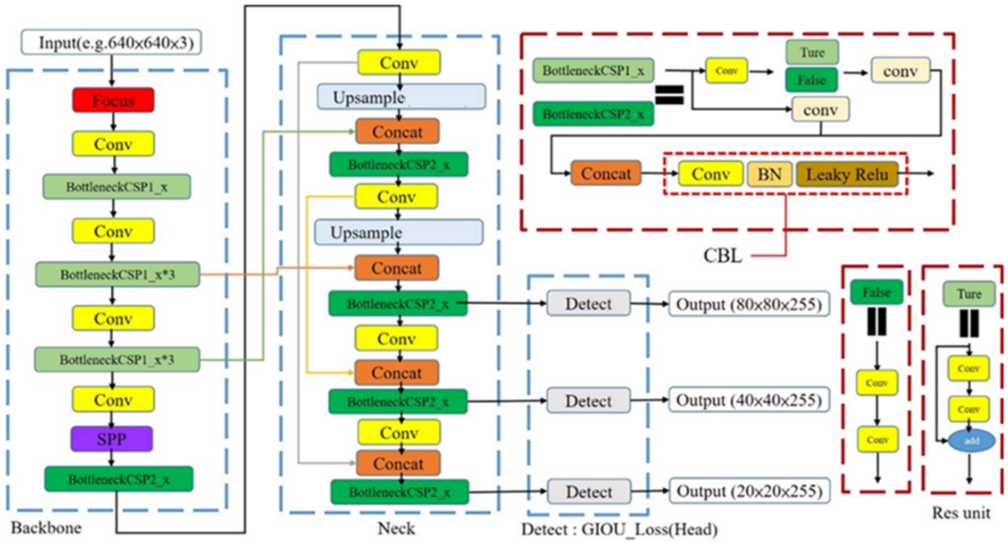

Figure S1. YOLOv5 network architecture

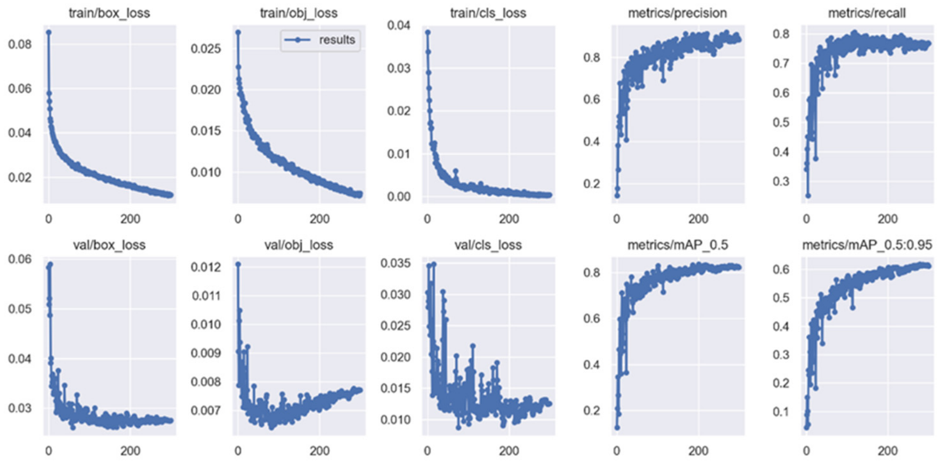

Figure S2. Convergence of loss functions for training set and validation set of original images and precision, recall, and average precision

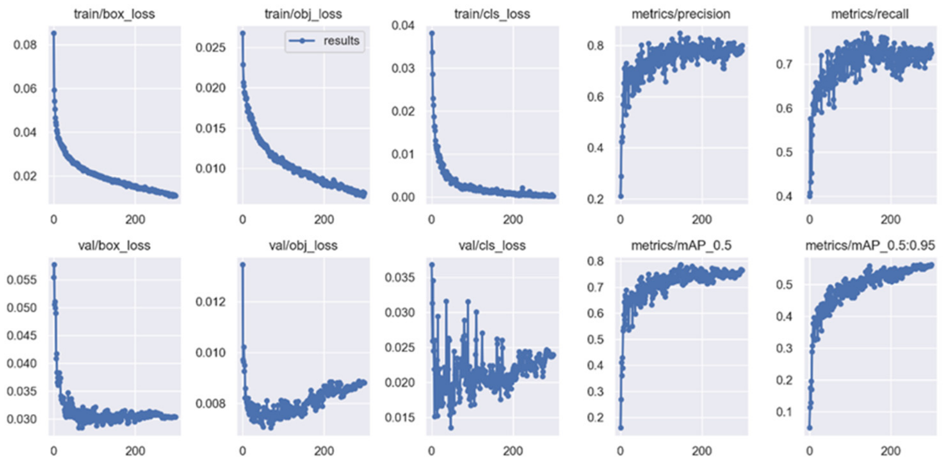

Figure S3. Hyperspectral image training set and validation set loss functions and convergence of precision, recall, and mean precision

S2. Equations for Classification Indicators

The sensitivity indicates how well the model can detect symptoms of esophageal cancer. The accuracy value indicates the proportion of esophageal cancer and actual cancer symptoms in the model's diagnosis. F1-score is a harmonic mean, and it can be used as a rough indicator of the model performance. Kappa value can be used to evaluate the consistency between prediction and pathological analysis results to evaluate the feasibility of prediction tools. Its value is between −1 and 1 and often has a threshold of 0.6. AP is a commonly used evaluation index for object detection, and the overall sensitivity and accuracy are used to quantify the overall performance of a prediction model.

$$\text{Precision} = \frac{\text{TP}}{\text{TP+FP}} \quad \text{(S1)}$$

$$\text{Sensitivity} = \frac{\text{TP}}{\text{TP+FN}} \quad \text{(S2)}$$

$$\text{Specificity} = \frac{\text{TN}}{\text{TN+FP}} \quad \text{(S3)}$$

$$F1 = \frac{2}{\frac{1}{Precision}+\frac{1}{Recall}} \quad (S4)$$

$$Accuracy = \frac{TP+TN}{TP+TN+FP+FN} \quad (S5)$$

Table S1. Heatmap of the confusion Matrix.

Results of the RGB Model				
TRUE				
	BCC	SCC	SK	Background FP
BCC				
SCC				
SK				
Background FN				
Result of HSI Model				
TRUE				
	BCC	SCC	SK	Background FP
BCC				
SCC				
SK				
Background FN				
